# Influence of the Characteristics of Expandable Graphite on the Morphology, Thermal Properties, Fire Behaviour and Compression Performance of a Rigid Polyurethane Foam

**DOI:** 10.3390/polym11010168

**Published:** 2019-01-18

**Authors:** Pablo Acuña, Zhi Li, Mercedes Santiago-Calvo, Fernando Villafañe, Miguel Ángel Rodríguez-Perez, De-Yi Wang

**Affiliations:** 1 IMDEA Materials Institute, C/Eric Kandel, 2, 28906 Getafe, Madrid, Spain; pablo.acuna@imdea.org (P.A.); zhi.Li@imdea.org (Z.L.); 2 Cellular Materials Laboratory (CellMat), Condensed Matter Physics Department, Faculty of Science, University of Valladolid, Campus Miguel Delibes, Paseo de Belén, 7, 47011 Valladolid, Spain; mercesc@fmc.uva.es (M.S.-C.); marrod@fmc.uva.es (M.Á.R.-P.); 3 GIR MIOMeT-IU Cinquima-Química Inorgánica, Faculty of Science, University of Valladolid, Campus Miguel Delibes, Paseo de Belén, 7, 47011 Valladolid, Spain; fervilla@qi.uva.es; 4 Department of Materials Science and Engineering and Chemical Engineering, Carlos III University, Avda. de la Universidad, 30, 28911 Leganés, Madrid, Spain; 5 Universidad Politécnica de Madrid, E.T.S. de Ingenieros de Caminos, 28040 Madrid, Spain

**Keywords:** polyurethane foam, expandable graphite, mechanical property, flame retardancy

## Abstract

Three types of expandable graphite (EG) differing in particle size and expansion volume, are compared as flame retardant additives to rigid polyurethane foams (RPUFs). In this paper we discuss microstructure, thermal stability, fire behavior, and compression performance. We find that ell size distributions were less homogeneous and cell size was reduced. Furthermore, thermal conductivity increased along with EG loading. Thermogravimetric analysis (TGA) showed that EG only increased residue yield differently. The results indicate that a higher expansion of EG increased the limiting oxygen index (LOI) value, whereas a bigger particle size EG improved the rating of the vertical burning test (UL94). Results from the cone calorimeter test showed that a bigger particle size EG effectively reduced peak of heat release rate (pHRR). Furthermore, a higher expansion, led to a decrease in smoke production (TSP). The combination of both characteristics gives extraordinary results. The physical–mechanical characterization of the EG/RPUF foams revealed that their compression performance decreased slightly, mostly due to the effect of a bigger size EG.

## 1. Introduction

Polyurethanes (PU) are a family of polymeric materials with a wide range of applications, including paints, adhesives, elastomers, and flexible or rigid foams [1]. Rigid polyurethane foams (RPUFs) are widely used in some industries like refrigeration, engineering, construction or electronics, since they are insulating materials with good mechanical properties [2,3]. However, RPUFs are also very flammable material, which is an important drawback for some of their final applications. Therefore, flame retardant additives are highly recommended in order to achieve a good flame retardant performance, to fulfil its practical demands [4]. Traditionally, halogenated flame retardants have been used, due to their superior smoke suppression and slowing flame spread characteristics. On PU foams, *tris*-(2-chlor-propyl)-phosphate (TCPP) was mainly used. However, environmental concerns related to the emission of toxic gases from the combustion of halogenated flame retardants have led the European Union (EU) requiring the development of new halogen-free flame retardants [5].

Different halogen-free flame retardants have been added to RPUF. Phosphorous compounds, such as toluidine spirocyclic pentaerythritol bisphosphonate (TSPB), can achieve a 26.5% in the limiting oxygen index (LOI) test from a TSPB content of 30% on a RPUF [6]. Other phosphorus compounds, such as 9,10-dihydro-9-oxa-10-phosphaphenanthrene-10-oxide (DOPO), and its derivatives, have produced good flame retardant performance, as well as better compressive strength [7]. Ammonium polyphosphate (APP) [8], dimethylmethylphosphonate (DMMP) [9], and ciclophosphazenes [10] have been also described as good solutions. Nevertheless, different inorganic flame retardants have shown even better results in terms of flame retardancy. Thus, the presence of aluminum trihydroxide (ATH) allows materials to reach a 72.5% in the LOI test. Although these results were obtained only with high loading of ~40 wt %, this seriously weakens the mechanical properties of the final foam [11,12]. Furthermore, expandable graphite (EG) is an inorganic flame retardant, which has received a lot of attention in recent years as a new halogen-free flame retardant [2,4,8,9,10,11,12,13,14,15]. EG is manufactured from flake graphite by treatment with strong acids like sulfuric or nitric acid, which are eventually intercalated into the graphite crystal structure, so that it expands or exfoliates when heated [16]. With the temperature increase, these intercalation compounds decompose into gaseous products, and produce a higher inter-graphene layer pressure. According to Camino et al. [17], the reaction of graphite with H_2_SO_4_ is a redox process, which releases CO_2_, SO_2_, and water. Thus, the volume of the graphite can increase up to 300 times, thereby lowering its density and increasing the surface area by ca. 10-fold. Once in the polymer, EG forms a worm-like insulation carbonaceous protective layer in the condensed phase with the polymer, thus isolating the material from heat and precluding mass transfer to heat sources [18,19]. The release of water and other volatiles also helps suffocate the flames [20]. 

The effect of adding EG on the properties of a water blown RPUF has been previously evaluated by several authors. Xiang-Cheng Bian et al. [21] described that higher density foams show better flame retardancy behaviors compared to lower density foams. In addition, the study of the mechanical properties showed a correlation between density and compressive stress [22]. EG not only makes the foaming process harder, but also worsens the mechanical properties of foam [9]. On the other hand, the effect of particle size and EG’s rate of expansion, were only studied separately. The effect of particle size of EG on semi-rigid polyurethane foams indicated that bigger particle sizes of EG gave better LOI, horizontal burning test, mechanical properties performance [23], and therefore a significant improvement of the thermal stability [24]. The rate of expansion was studied by Lorenzetti [25], who showed that the rate of expansion had no significant influence on flame retardancy performance, playing only a limited role on the LOI test, and no effect on the cone calorimeter test. On the contrary, Xiao-Liang Zhang et al. [26] indicated that the efficiency of EG depends on its expandability; the more expandability, the better flame-retardant performance. Thus, no conclusion may be drawn about which parameter dominates the mechanism of EG in order to achieve a precise flame retardant behavior.

The purpose of this research is to study the effect of several EGs with two different particle sizes; 300 and 500 µm, as well as different expansion volumes; 250 and 350 cm^3^/g, on the properties of RPUFs. Both particle size and expansion volume were studied, taking into consideration the impact on the flame retardancy and thermal stability. Indeed, cellular structure, density, and compression properties of EG/RPUF composites were also reported and discussed based on the different particle sizes of EG. Besides, the divergence of some previously reported results, and the absence of systematic investigations regarding the optimization of EG performance, encouraged us to develop this study. We aimed to select the best characteristics of EG as a flame retardant additive for RPUFs, and to find a balance between its flame retardant and compression performance. In this paper, three types of EG (EG1, EG2, and EG3), with different contents (0, 6, 8, and 10 wt %) were used as flame retardant additives to RPUF. 10 flame retardant RPUFs were prepared. The influence of particle size, expansion volume, and filler content on foam properties are herein systematically discussed. 

## 2. Experimental 

### 2.1. Materials

Polyurethane raw materials FOAM-IT!™ 5 (PU) were provided by Smooth-On (FormX Spain, Barcelona, Spain): polyurethane raw materials were divided in two sides: A-side (referred to isocyanate), and B-side (referred to polyol, containing catalysts, surfactants, and water as blowing agent). For the RPUF, the manufacturer provided the following data: free rise density, 80 Kg/m^3^; viscosity, 300 cps; and mixing ratio, 100/87 (A:B). Expandable graphite (EG), flame retardant GHL PX 95 N (Ash max. 5%, moisture 1%, volume expansion of 250 cm^3^/g, starting temperature 180–220 °C, and 300 µm particle size; EG1), GHL PX 98 HE (Ash max. 2%, moisture 1%, expansion volume of 350 cm^3^/g, starting temperature 180–220 °C, and 300 µm particle size; EG2), and GHL PX 99 32 350 (Ash max. 1%, moisture 1%, expansion volume of 350 cm^3^/g, starting temperature 150 °C, and 500 µm particle size; EG3), were kindly provided by Georg H. Luh, GmbH (Walluf, Germany).

### 2.2. Foam Preparation

RPUFs with 0, 6, 8, and 10 wt% EG were prepared using the one-shot and free-rising methods. The B-side parts of the RPUFs and EG were firstly mixed in a plastic beaker for 2 minutes with a mechanical stirrer at 1000 rpm until uniform dispersions were obtained. Then, the correct amount of isocyanate (A-side) was added, and the mixture was stirred rapidly at 1200 rpm (for 30 s until the system became homogeneous) with a heidolph RZR-1 mechanical stirrer. The mixture was finally poured into a 250 mm × 250 mm × 60 mm open aluminum mold, where the RPUFs were raised in vertical direction at room temperature. After curing at room temperature for a minimum of 3 h, samples were cut according to the standards of the different test performed. RPUFs formulations are shown in Table 1 below.

### 2.3. Characterization 

Foam density was measured as described by ASTM D1622/D1622M-14 [27]. Density was determined by dividing the weight of each sample by its corresponding volume. Three different cylindrical samples of 30 mm × 30 mm (diameter × height) for each material were measured. Their data and average standard deviations were reported.

The percentage of open cells (OC %) was measured using a gas pycnometer Accupyc II 1340 from Micromeritics, according to ASTM D6226-10 [28]. Open cell content was measured for three cylindrical samples (with the same dimensions of those used for density measurements) from each material after measuring their densities. Their data and average standard deviations were reported.

The cellular structure of the foam and SEM micrographs were determined by Scanning Electron Microscopy (SEM) with a JEOL JSM-820 microscope (JEOL, Tokyo, Japan). The cured foams were cut in order to ensure a smooth surface, which was examined by SEM after vacuum coating with a gold monolayer. SEM micrographs were obtained for the growth plane (z plane) of the foam. An image analysis technique [29] of the SEM micrographs was used to determine the main characteristics of the cellular foam structure: cell size distribution, mean cell size (Φ_3D_), and anisotropy ratio (AR). The calculated statistical parameters for cell size distribution were: standard deviation (SD), normalized standard deviation (NSD), and asymmetry coefficient (AC). The experimental uncertainties for cell size were ~5% and the experimental uncertainties for the NSD and AC were ~8%. These experimental errors were obtained by the differences when measuring the same parameters again using other micrographs of the same samples, and repeating the whole process of image analysis.

Thermal conductivity was determined at ca. 25 °C using a transient plane source thermal constant analyzer, Hot-Disk TPS 2500S, according to ISO 22007-2:2008 method [30]. The measurements were performed using two cylindrical samples cut from the same materials of 30 mm × 30 mm (diameter × height). A disk-shaped TPS sensor (Hot Disk, Gothenburg, Sweden) with a diameter of 3.189 mm was used in all measurements, after being located in contact with the xy plane (perpendicular to growth plane) of the two samples [31]. Five measurements were obtained for each material. Their data and average standard deviations were reported.

The thermal stability of the specimens (TGA) was studied by a Q50 thermogravimetric analyzer (TA instruments, New Castle, DE, USA), under nitrogen atmosphere, at a heating rate of 10 °C/min from 30 to 700 °C using ca. 10 mg of each sample, placed in a platinum cup.

The limited oxygen index (LOI) test was performed with an oxygen index test instrument (FTT, East Grinstead, UK) following the ASTM D2863-13 standard [32]. The size of the specimens measured was 130 mm × 10 mm × 10 mm (length × width × thickness).

The UL94 vertical burning test was performed on an UL94 Horizontal/Vertical Flame Chamber (FTT, East Grinstead, UK) according to ASTM D3801-10 standard [33]. The height of burning flame was 20 mm. The size of the samples measured was 130 mm × 13 mm × 10 mm (length × width × thickness). Five specimens were tested for each sample. Their data and average standard deviations were reported.

The cone calorimeter test was performed with a cone calorimeter instrument (FTT) following ISO 5660-1 standard [34] under an external flux of 50 kW/m^2^. The samples of 100 mm × 100 mm × 30 mm (length × width × thickness) were wrapped in aluminum foil, and exposed horizontally to the external flux. Measurements were made in duplicate. Their data and average standard deviations were reported.

Compressive properties were tested with an INSTRON 566 instrument following ASTM D1621-00 standard [35]. A maximum of six specimens for each sample, with size 50 mm × 50 mm × 30 mm (length × width × thickness), were tested. The compressive constant rate was 3 mm/min. The properties were measured in the direction of foam growth. The average compressive strength and average standard deviations were reported.

## 3. Results and Discussion

### 3.1. Cellular Structure Analysis and Thermal Conductivity

The main characteristics studied for the cellular foam structure were: open cell content (OC %), mean cell size (Φ3D), cell size distribution, anisotropy ratio (AR), density, and thermal conductivity. Furthermore, the statistical parameters for cell size distribution determined were: standard deviation (SD), normalized standard deviation (NSD), and asymmetry coefficient (AC). NSD (ratio between the SD and Φ3D) is related to the width of cell size distribution, and therefore provides information about the homogeneity of the cell size distribution. Thus, homogeneous cell distributions present small values of NSD, whereas AC provides information about the shape of the distribution. All these parameters are collected in Table 2. 

Density is one of the most critical parameters of a RPUF, because it affects the distribution of the filler (EG) in the polymeric matrix, and so its impact on density determines its performance for many applications [15,23] such as the strength, modulus, and energy absorption ability of the foam [32,36]. EG-filled RPUF showed higher density than those of neat foam. This may be related to the increase of the polyol viscosity when EG was incorporated into the RPUF matrix, along with the very high density of EG particles cooperated to the RPUF matrix (for example, 2.2 g/cm^3^ for EG1 versus *ac.* 0.1 g/cm^3^ for neat PU foam). However, the three different particle sizes of EG impacted the RPUF´s density differently. As an example, EG1 was the filler which produced a higher increase over this parameter. Thus, an increase on average of 8.0% was observed with the addition of EG1, of 4.8% with EG2, and of 4.9% with EG3. Considering the foams containing 10 wt % EG, the density grew by 9.4% for EG1 samples with respect to that of neat foam, by 6.9% for EG2, and by 5.9% for EG3. As a matter of fact, this phenomena was related to the impact of smaller EG particle size on the neat foam, by decreasing the average cell size due to a nucleating effect during the foam formation, as reported by Luo et al. [24]. 

One decisive parameter in RPUFs is open cell content (OC %), since closed cell structures were required for structural and thermal insulating applications. A slight increase in open cell content compared to that of reference foam (6.90%) was observed with the addition of EG particles. However, open cell content was similar for all different EG particles (between 9.1% and 9.9%), except for the foam containing EG3 at 6 wt %, which was the highest (10.8%), and for the foam containing EG1 at 8 wt % (7.8%), which had the lowest value for the EG-filled materials. This indicated that EG, as a filler, increased the OC %; mostly, bigger EG particles increased the OC % in a greater way. 

**Figure 1:**
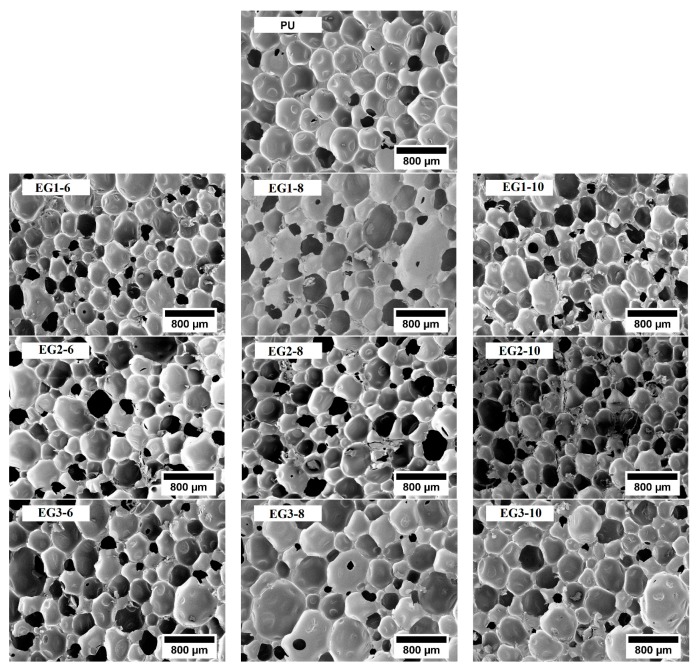
SEM micrographs of the growth plane of rigid polyurethane foam (RPUF) samples.

**Figure 2:**
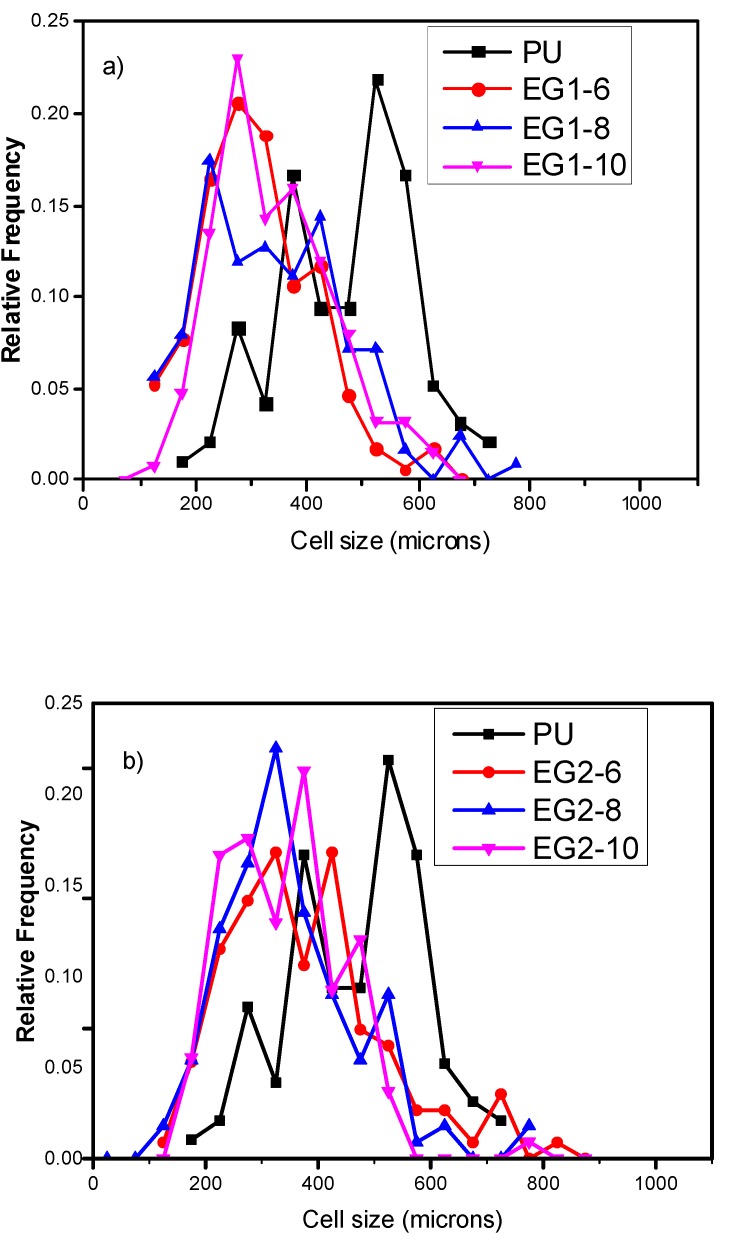

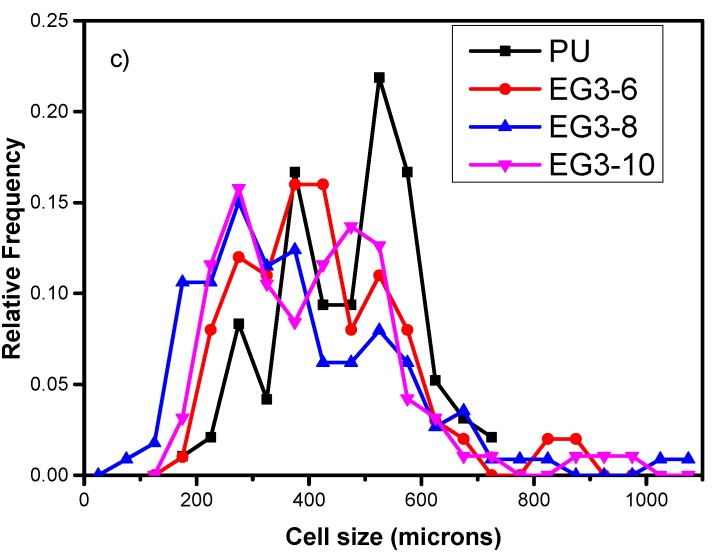
Cell size distribution for (**a**) EG1, (**b**) EG2, and (**c**) EG3 RPUF samples.

**Figure 3:**
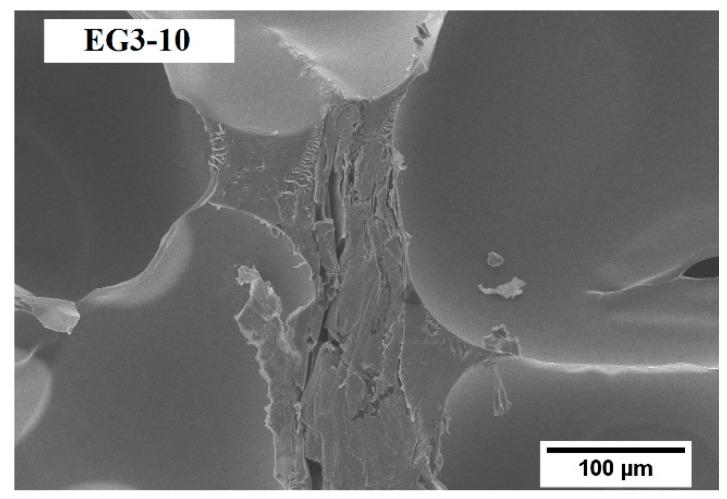
SEM micrograph of EG3-10 sample in the RPUF matrix.

Cell size is a crucial factor influencing the properties of foams. The analysis of RPUF samples for SEM micrographs is shown in Figure 1. As previously described in other studies, EG particles have a nucleating effect during foam formation, decreasing the average cell size [4,37]. In particular, the addition of EG1 promoted the highest cell size reduction (34% for the sample with 6 wt % content), whereas the average cell size reduction reached 30% for EG1, 27% for EG2, and 13% for EG3. Therefore, the highest cell size reduction was obtained for EG particles with the lowest size (300 µm), due to the aggregation between particles [24]. Larger particle size EG (EG3) enlarged the cell sizes (500 µm), which was probably located mainly in the struts, that is, the intersection of several cells. On the other hand, the lower particle size EG (EG1 and EG2; 300 µm) were not located there due to the lower size compared with the neat foam (467 µm) [23]. Instead, the anisotropy ratio (AR) of loaded EG/PU did not show significant changes with respect to that of neat PU. On the other hand, the histograms depicted in Figure 2 provide valuable additional information about cell size distribution. The data indicated that the presence of EG particles modified cell size distribution, increasing the heterogeneity (NSD values rise) and asymmetry (AC values move away from zero). The distribution was more asymmetric with the highest particle size EG (EG3). The size of the EG particles used (300 or 500 µm) was in the same order as the mean cell size of foam, which might be the main reason behind EG particles being located between cell walls and struts [37] (Figure 3), thus modifying cellular structure. 

One of the main applications of RPUF is their use as insulating material, due to their low thermal conductivity, which is well represented as the sum of gas phase, solid phase, and radiative conductivity [38]. Therefore, thermal conductivity of RPUF was herein measured in order to evaluate the effect of EG. Indeed, these thermal conductivity measurements were made once the foam reached a stationary state, that is, when the carbon dioxide inside the cells (generated during the foaming reaction) was completely substituted by atmospheric air (approximately two weeks after production) [38]. It is well known that particles in the RPUF formulation can act as nucleating agents, decreasing cell size and/or acting as infrared radiation blockers, which increases the extinction coefficient. Consequently, this implied a decrease in radiation contribution to thermal conductivity. However, thermal conductivity increased with the addition of increasing amounts of EG particles (Table 2), which could be explained considering the increase in conduction through the solid phase, due to the following reasons: the high density (around 100 kg/m^3^) of PU formulations used in this study [39,40], the increase in foam density due to the EG addition [38], and the formation of a network for conducting interconnected EG particles throughout the insulating PU matrix for higher EG contents (above six wt %) [41]. Finally, the addition of EG particles slightly increased the open cell content in the foams, which contributed to increased thermal conductivity.

### 3.2. Thermogravimetric Analysis

**Figure 4:**
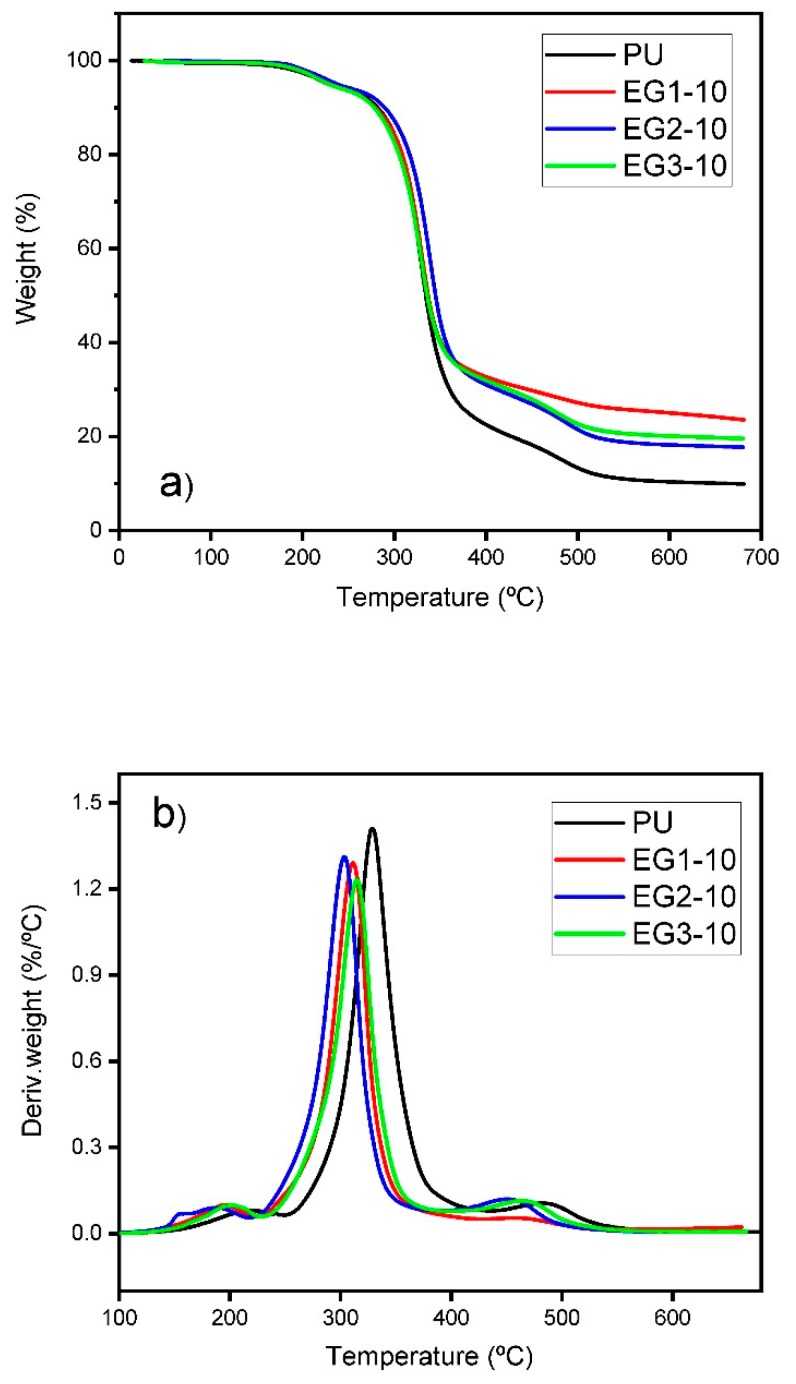
(**a**) TGA and (**b**) DTG curves for RPUF samples containing EG1, EG2, and EG3 at 10 wt % loading.

TGA is one of the most relevant analysis for evaluating thermal properties of RPUFs. Thermogravimetric analysis (TGA) and differential thermogravimetry (DTG) analysis of neat RPUF (PU) and EG-filled RPUF samples on N_2_ atmosphere are illustrated in Figure 4 (samples containing 10 wt % of EG) and S1, the data is collected in Table 3. The corresponding thermal parameters were: the initial decomposition temperature (T_5 wt %_, namely the temperature at 5% weight loss), the temperature at maximum weight loss rate (T_max_, denoted as the peak value from the DTG curves), and the remaining residue at the end of the test. Both unfilled and EG-filled RPUFs showed a similar degradation curve for each sample. The degradation process consisted of a 2-steps process [36,42]. The first step of degradation for the neat RPUF was between 150 and 250 °C with *ca*. 7% weight loss. This step may be associated to the evaporation of small molecules of unreacted isocyanate monomers [43]. The second step of degradation occurred between 250 and 620 °C, and it was mainly due to the formation of the original PU components (isocyanates and polyols), due to the break of urethane bonds in hard segments [37,43]. Isocyanate formed remains, but polyol segments also decomposed into aliphatic ether alcohol, olefins, and CO_2_. As the temperature increased, more complex products were formed, such as amino compounds and CO_2_ from former isocyanate were removed at *ca*. 350 °C. At higher temperatures (*ca.* 500 °C), other products derived from isocyanate groups (such as amines or benzene alkyl) decomposed into volatile products like CO_2_, HCN or NO_2_, which were generated in the final stage of the degradation process [42]. 

For the EG-filled foams, the degradation started earlier. Some authors have attributed the earlier degradation temperature of foam to the oxidation and degradation of polymer matrix promoted by the residual acid inside the layers of EG [17,24,44]. In fact, the average of the first peak degradation temperature on the DTG curves for the EG1 samples (with lowest rate of expansion) was 194 °C. For EG2 and EG3 samples (both EGs with the same rate of expansion), they increased to 202 and to 201 °C, respectively. In other words, the higher rate of expansion, delayed the degradation starting point. For EG1, the average of the first step of degradation, not taking into account the loading, was in the range 121–223 °C, whereas it was 132–233 °C for EG2, and 136–228 °C for the particles with higher size EG3. The increasing range could be explained considering the higher rate of expansion combined with a bigger particle size of EG particles, which delayed the degradation path mechanism once the acid has been volatized. The average of the maximum degradation temperature (T_max_) for EG1 was 310 °C, whereas it increased to 317 °C for EG2, and was slightly reduced to 315 °C for EG particles with higher size; EG3. Meanwhile, the residual char increased for all EG-filled RPUF with respect to neat PU. The residue wt % increased along with the EG loading to reach a maximum of 22.6 wt % for EG1. The char layer formed acted as a thermal barrier, protecting the foam from further decomposition and limiting the degradation of the polyurethane matrix. Higher rate of expansion did not improved the char residue, neither did a higher particle size. A turning point regarding particle size and rate of expansion was found for EG1 samples, and they were the best according to the char residue wt %. According to Yi Li. et al. [37], bigger particle size of EG did not influence the T_max_, but in accordance with the high rate of expansion, more gases can be generated, resulting in less residual char. Although the rate of expansion increased the degradation temperature, only minor influences were observed. Considering the effect of EG in the foam, except for the residue wt %, there were no important impacts on the polyurethane degradation, as EG did not react with the polyurethane matrix [45]. 

### 3.3. Fire Behaviors 

#### 3.3.1. LOI and UL 94 Tests

LOI and UL94 tests were performed, and the corresponding results are collected in Table 4. Indeed, in order to compare the RPUF specimens, total burning time—the time in which the five specimens from the UL94 tests were burning until being total extinguished—was herein recorded and presented. The LOI of neat RPUF (PU) was 19.2%, indicating that it was a very flammable material under atmospheric conditions (*ca*. 21% O_2_). LOI values and UL94 rating increased along with the increasing amount of EG into the foam. A V-0 rating in the vertical burning test was only achieved above 8 wt % onwards on the EG1-RPUF sample, which provided a LOI value of 27.8%. The sample containing 10 wt % of EG1 increased by a 50% the initial value of the PU to 29.8, whereas UL94 burning total time decreased (20.9 s) and maintained the V-0 rating. When EG1 was replaced by EG particles with a higher rate of expansion; such as EG2 (350 cm^3^/g), the effects on the LOI values were positive. LOI increased from 27.8% to 30.0% in EG2–8 and higher LOI values were achieved using 10 wt % of EG-2, showing a value of 31.8%. UL94 rating of the EG2–8 and EG2–10 samples was indeed V-0. In spite of the increasing the total time for UL94 test, the performance of EG2 was almost similar to EG1 on the UL94 test. So, the rate of expansion of EG, had no big influence on the UL94 test, maintaining the same rating. Moreover, EG3, with higher particle size and same rate of expansion as EG2, obtained similar LOI performance. The samples’ LOI values were clearly improved with the increasing rate of expansion, which might be explained by the formation and densification of an isolation layer that became larger with increasing volume of the EG particles and formed a more intumescent char [23,25]. On the contrary, decrease in particle number (caused by the higher particle size for the same loading) had no important effect on the formation of a compact isolation layer during the fire process, increasing slowly the LOI values as it was reported by other authors [23,25]. Interestingly, the UL94 values obtained by EG3-RPUFs were outstanding. In fact, a V-1 rating was achieved with a loading as low as 6 wt % of EG3, whereas for 8 wt % onwards, the UL94 rating was V-0. This behavior may be explained considering the carbonaceous char layer under the polyurethane surface, which protects the polymer from the flame on these tests. Therefore, an increasing amount of EG added into the foam should contribute to an increased barrier effect due to EG expansion, giving eventually a good fire performance [17,18,19,20]. For this reason, EG3—the EG particles with higher size—should be able to create a more compact isolation layer, thus improving UL94 performance [24,46]. In summary, the role of particle size and rate of expansion of EG, as intrinsic characteristics of the material, seemed to be crucial for the flame retardancy performance of EG-filled RPUFs. On one hand, the expansion rate was important for a variable O_2_ % test as LOI, in which a higher concentration of O_2_ could easily start a fire process. On the other hand, the particle size of EG3 was determined on the vertical burning test under ambient conditions in order to prevent fire spreading in a more efficient way [24]. 

#### 3.3.2. Cone Calorimeter Test

**Figure 5:**
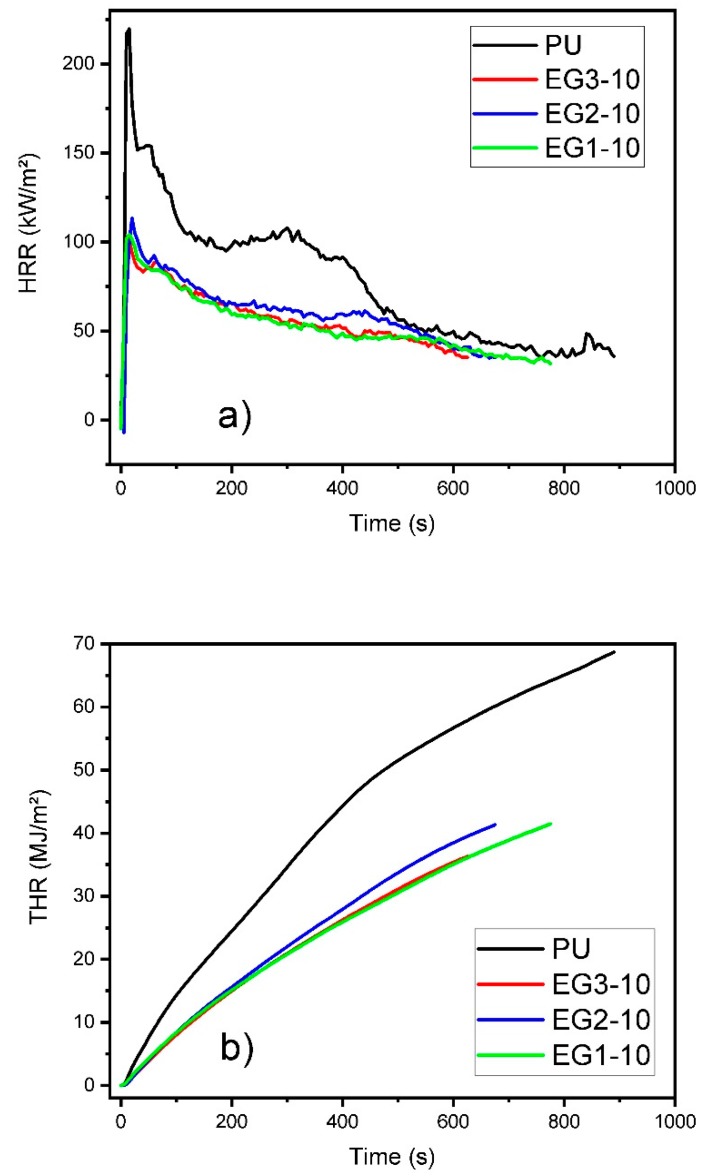

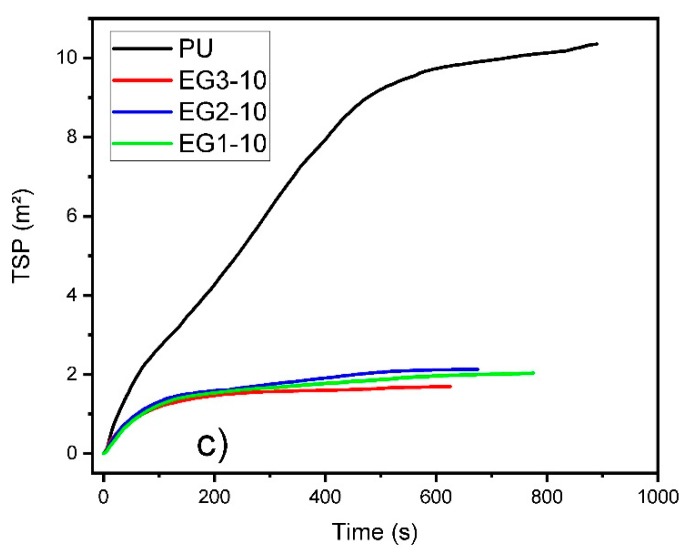
(**a**) Heat release rate (HRR), (**b**) Total heat released (THR), and (**c**) Total smoke production (TSP) curves for RPUF samples containing EG1, EG2, and EG3 at 10 wt % loading.

The cone calorimeter test has been extensively used to investigate the fire hazard of polyurethane matrix foams when subjected to heat flow, since this test correlates well with large-scale fire tests and simulates a real fire scenario [47]. This experiment was based on the oxygen-combustion principle test, and was performed at a heat flux of 50 kW/m^2^, thus giving a quantitative analysis, since it provides different characteristic parameters such as pHRR (Peak Heat Release Rate), TSP (Total Smoke Production), TSR (Total Smoke Release), THR (Total Heat Released), and mass loss rate (Mass %). All these data for the RPUFs are summarized in Figure 5 (at 10 wt % loading of the EGs) and Table 5. Each RPUF loaded with different EG types was discussed individually.

The pHRR and TSP curves for the PU foams loaded with EG1 are shown in Figure S2, whereas their THR and mass loss curves are displayed in Figure S3. pHRR is the most important parameter for fire safety evaluation, since it indicates the burning behavior of materials that form residues after the burning process [48]. It is noteworthy that the samples rapidly raised their maximum value (pHRR) after ignition. The reason behind this phenomena was their porous structure, which facilitated a large surface contact area between material and air, currently being more exposed to heat [49]. For instance, neat RPUFs burned fiercely once ignited and exhibited a peak value of 220 kW/m^2^. Two peaks were detected on these samples. The first peak may be attributed to the development of an intumescent protective char layer, and the second peak should be mainly due to the degradation of this layer to heat exposure [50]. Thermal feedback may also contribute to this second peak, when the pyrolysis front approaches the insulated back surface of the sample [25,48]. The maximum values of these second peaks decreased as the amount of EG increased, since pyrolysis at the samples’ bottom parts was then blocked [24,25]. Therefore, the addition of EG1 reduced pHRR values by 32%, 39%, and 49% for the samples containing 6, 8, and 10 wt % of EG, respectively, with the lowest peak for the EG1-10 samples being 111 kW/m^2^. TSP is a critical parameter, since smoke is a dangerous agent on a fire scenario, and mainly due to the toxicity of CO. Hence, reducing the TSP or TSR evidenced a good flame retardant effect. TSP of neat PU showed a value of 10.4 m^2^, and the addition of EG continuously reduced the TSP reference value: they were reduced by 25%, 63% and 81% in the samples containing 6, 8 and 10 wt % of EG respectively, with the lowest value for the EG1-10 sample being 2.0 m^2^. 

THR curves for RPUFs loaded with EG1 are shown in Figure S3. THR of the reference sample (PU) gave a value of 68.7 MJ/m^2^. The introduction of EG continuously reduced this value by 18%, 34%, and 27% for samples containing 6, 8, and 10 wt %, respectively of EG1, with the lowest value for the EG1-8 sample being 45.3 MJ/m^2^. The mass loss rate is another important parameter in order to evaluate the flame retardant properties of a polymer. Commonly, neat polymers made from petroleum derivatives are very flammable, therefore flame retardant treatments are mandatory, and this parameter must be improved. In our case, mass loss of the neat PU sample showed a value of 87 wt %. In this way, most polymers were transformed into volatiles during the process. Significantly, the addition of EG reduced this reference value by 17%, 28%, and 33% for samples containing 6, 8, and 10 wt % of EG, respectively, the lowest mass loss for the EG1-10 sample being 54 wt %. For our EG-filled RPUFs, increasing quantities due to the EG expansion led to the formation of a carbon char when exposed to a heat source, thus improving the polymer flame retardancy. As concluded previously by other scientists, the intumescent char formed by the EG expansion contributed flame suppression, and limited heat and mass transfers from the polymer to the heat source, thereby preventing further decomposition and limiting RPUFs’ weight loss [2,24,37,50].

pHRR and TSP curves for RPUFs loaded with EG2 are shown in Figure S4. THR and mass loss are plotted in Figure S5. As well as EG1 samples, EG-filled RPUF samples reached their maximum peak very rapidly; less than 20 s. Thus the time of EG2-10 increased by 5 s compared to that of EG1. Alike, the addition of EG reduced the pHRR value of neat PU continuously by a 25%, 42%, and 48% in the samples containing 6, 8, and 10 wt % of EG2, respectively, with the lowest peak for the sample with EG2-10 being 113 kW/m^2^. In this way, the addition of EG2 reduced continuously the reference TSP values (PU) by a 52%, 79%, and 79% in the samples containing 6, 8, and 10 wt % of EG2, respectively. Then, 2.1 m^2^ was shown as the lowest value for the sample with EG2-10. 

THR and mass loss curves for RPUF containing EG2 are shown in Figure S5. The introduction of EG2 also reduced the THR value continuously by a 29%, 43%, and 40% in the samples containing 6, 8, and 10 wt % of EG2, respectively, with the lowest result for the sample EG2-8 being 38.9 MJ/m^2^. The addition of EG2 also reduced the reference value of mass loss up to a 34% reduction on EG2-10. So far, 54 wt % was the lowest amount of mass loss during the experiment for the sample EG2-10. 

pHRR and TSP for the RPUFs loaded with EG3 are shown in Figure S6. THR and mass loss are displayed in Figure S7. As described above for previous samples, PU containing EG3 again reached their pHRR before 15 s, in the same range as other EGs. In this case, the addition of EG also reduced the reference pHRR value continuously by a 43%, 51%, and 54% for the samples containing 6, 8, and 10 wt % of EG3, respectively, with the lowest peak for the sample with EG3-10 being 101 kW/m^2^. Thus, the addition of EG3 also continuously reduced the TSP reference value by a 67%, 82%, and 84% for samples containing 6, 8, and 10 wt % of EG respectively. In this case, an impressive 1.7 m^2^ was the lowest value for the EG3-10 sample. 

THR and mass loss curves for RPUFs containing EG3 are shown in Figure S7. Again, the introduction of EG3 reduced the reference value by 18%, 47%, and 47% for the samples containing 6, 8, and 10 wt % of EG3, respectively, with the lowest result for a sample with higher EG wt % as EG3-10 being 36.3 MJ/m^2^. Therefore, the addition of EG3 also reduced the reference value of mass loss by 18%, 39%, and 37% for the samples containing 6, 8, and 10 wt % of EG3, respectively. The lowest amount of mass loss during the cone calorimeter test for the sample with 8 wt % EG3 was 49 wt %.

In order to clarify which intrinsic characteristics of EG were decisive for its performance as flame retardant, an analysis including the impact of particle size and rate of expansion on the flame retardant performance was carried out. Thus, the effect of rate of expansion could be studied by comparing EG1 and EG2, bearing in mind that these two types of EG had the same particle size at different rates of expansion (250 and 350 cm^3^/g, respectively). The pHRR at the maximum difference point was 6% lower for EG2, which had a higher rate of expansion. Afterwards, THR of samples with a 10 wt % of EG was 18% lower for the foams with higher expansion rates, although it was a 15% lower considering the average values. Owing to the rate of expansion of EG2, THR was lower. One possible explanation could be that the heat could not be transferred so easyily trough a higher volume material after the expansion mechanism of EG. In this case, a higher amount of PU remained unaltered in the condensed phase, which was not available for pyrolysis, which eventually reduced THR [25]. On the other side, EG2 reduced TSP and TSR at higher rates than EG1, with a higher reduction of 43% lower than EG1 when an 8 wt % EG-2 was used. On average, the amount of smoke was 24% lower for EG with higher expansion. Interestingly the above difference between EGs decreased with an increase in EG loading. At last, the mass lost during the cone calorimeter test was slightly lower for EG2, although no big differences were found. All in all, these results support that EG2 was a better flame retardant than EG1. The above mentioned higher performance could be explained considering the fact that a higher expansion of EG promoted the development of a thicker worm structure, which can block more efficiently the heat and mass transfer during the pyrolysis, leading to lower values of pHRR, THR, and smoke production [25,37]. It can be concluded that the higher rate of expansion of EG2, has a very positive effect regarding the THR, mostly the TSP and TSR, where the differences were highlighted at lower loadings of EG, with a higher effectivity at higher rates of EG expansion.

On the other hand, to study the effect of particle size on flame retardancy, the behavior of EG3 and EG2 was compared next, taking into consideration that particle size increased from 300 to 500 µm, whereas the rate of expansion remained unaltered (350 cm^3^/g). Commonly, bigger particle sizes and expansion rates lead to higher flame retardant performance [51,52,53,54]. Consequently, the pHRR of EG3 samples was 24%, 15%, and 11% lower than that of EG2 samples containing 6, 8, and 10 wt % of EG3, respectively. In other words, particles with higher size were able to reduce more efficiently the pHRR. Considering the average values, the decrease of pHRR for EG2 was 17% lower than that of EG3. Even so, when the amount of EG increased, this difference was continuously reduced. In a similar trend, EG3 also reduced the smoke production (TSP) and the smoke released (TSR) at higher rates than EG2. In fact, a 30%, 13%, and 20% respective reduction with respect to EG2 was obtained for samples containing 6, 8, and 10 wt % of EG3. This result indicated that particles of higher sizes formed a more stable char barrier, which blocked the smoke production, as described by others [23,24,37]. On the other hand, THR of the sample with EG3 was higher for those samples containing 8 wt % of EG or less. Even though particles of higher sizes favored char barrier formation, bigger cracks could occur at the surface, thus reducing the effect of heat blocking and increasing THR as consequence [37]. Moreover, the thermal conductivity increased with the average cell size; as reported on the morphology analysis above; and with the breaking of the cell walls [23]. Finally, when comparing the mass loss rate of both samples, those containing EG3 improved the mass loss rate rather than those with EG2. This was due to the increasing number of particles forming a char barrier, and thus forming a more compact isolation layer [37]. For this reason, the heat flux delivered to the pyrolysis front of the sample was also reduced [25]. On the contrary, at 6 wt % of EG3, a higher amount of gases could be generated, as the small number of particles could not form a sufficient barrier to hinder heat penetration [37]. Therefore, the defects in structure also became larger, resulting in a less stable structure, and producing less amounts of residual char [24]. When the loading increased, the char became more compact and the initial PU structure remained almost unaltered, reducing the mass loss rate along with the aforementioned parameters. Therefore, the combination of higher particle sizes and higher rates of expansion had a beneficial effect on the formation of a compact residual char, which was the main characteristic of the mechanism of EG as flame retardant [14]. In summary, the higher the particle size, the bigger impact on the results of the cone calorimeter test, such as pHRR and THR, mostly TSP and TSR. At the same time, a higher rate of expansion was a necessary characteristic of this flame retardant to obtain an outstanding performance.

### 3.4. Mechanical Properties

#### Compression Test

The introduction of fillers in a polymeric matrix usually has important effects on the compression properties of foam [22]. Thus, compression tests are needed to completely characterize the foams. Herein the compression tests were performed at a constant rate of 3 mm/min, the data obtained are shown in Figure S8, and collected in Table 6. 

The compressive strength of the RPUF decreased slightly with the addition of EG, as well as in accordance to the EG loading; from 1% to 9%. The average compressive strength of EG1 decreased by 6% compared to neat foam, whereas EG2 and EG3 decreased by a 3% and 4%, respectively. A very interesting effect of EG was found at lower loadings (6 wt %), where a correlation at 6 wt % EG was observed between the cell size and the compressive strength. Thus, the lowest cell size obtained (EG1-6), gave the lowest compressive strength result. In addition, the highest cell size obtained (EG3-6), gave the highest compressive strength result. This effect was associated to the higher homogeneity of EG-filled RPUF at lower loadings, where the effect of particle size was more important on cell size and compressive strength due the homogeneity of cell size distribution, thus highlighting the differences. However, higher loadings of EG were associated with an increase in cell diameter, significant cell rupture, collapse, and brittleness, since EG particles can go through the cells damaging their structure [3,37], thus reducing the crosslinking density of the polymer matrix [41]. In summary, the mechanical performance worsened, even though density increased due to the addition of EG particles. All these results were in accordance with the previous findings of Thirumal et al. [22,23] and Modesti et al. [55]. Specifically, the compression performance of the RPUF loaded with EG3-–10 decreased by a dramatic 15% compared to neat foam. Smaller EG particles formed aggregates due to poor dispersion, thus decreasing the compression performance at higher rates [4,23]. Besides, the increase in EG size particles caused larger defects in the PU structure, as a result of a poor interfacial adhesion between EG and the PU matrix. For that reason, slippage among these two components took place, resulting in a bigger decrease in the compressive performance, which was in accordance with previous reports by Yi Li et al. [37]. The large particle size of EG could damage cell walls, since they tend to be placed inside cell struts, what increase mean cell size [25]. Therefore, the EG/RPUF structure became inhomogeneous, and consequently the compression performance was slightly worse than that of neat RPUF [4,13,56].

## 4. Conclusions

Rigid polyurethane foam-based composites were produced by adding different EG particles differing in size and rate of expansion. The effect of the aforementioned characteristics of EG attending to the cellular structure, thermal stability, fire behavior, and mechanical properties was studied. The introduction of EG particles into a RPUF matrix modified the cellular structure of foam, where the larger EG particles were those making the foam’s cell size distribution less homogeneous. Density increased for all EG types and loadings. The open cell content slightly increased with EG addition, and cell size increased along with the size of EG particle. The inclusion of different EG particles diminished the thermal insulating properties of foams, principally due to the increase in conduction through the solid phase of thermal conductivity. TGA analysis may be interpreted by considering a 2-steps process: RPUF degradation by an acid catalyzed procedure, and large improvement of the char residue of foam. It was not found to have a big impact on the thermal degradation due to the intrinsic characteristics of EG. Flame retardant properties were clearly improved by the loading of EG particles. LOI was improved to an impressive 31.8% when 10 wt % EG3 was used. The same result was achieved with EG2, which had the same rate of expansion, but lower particle size. LOI was also increased by the decrease in particle number caused by the increasing particle size. UL94 test gave outstanding results for the RPUF loaded with EG3, as it achieved a V-1 rating when only containing 6 wt % EG3. Furthermore, a V-0 rating was achieved for further loadings of EG3. On the other hand, 6 wt % EGs with 300 µm particle size, could not be classified. The higher particle size was able to create a more compact isolation layer, lowering the burning speed. Cone calorimeter tests showed that the presence of EG decreased pHRR, THR, TSP, and mass loss rate, being EG3 the most effective at all parameters studied. All in all, the better flame retardant performance was obtained for particles with higher size and hrate of expansion. All the same, particle size was believed to be the critical parameter behind this outstanding performance, taking into account all the flame retardant tests. Compressive strength tests showed that EG with the lowest particle size slightly diminished the compressive performance, whereas, a higher particle size led to a greater decrease. Although the increasing density had a positive influence on the compressive strength, the poor interaction between EG and the PU matrix leads to poor compression performance. 

## Figures and Tables

**Table 1:** The formulae of rigid polyurethane foams samples (RPUFs).

Samples	EG content (wt %)
PU	0
EG1-6	6
EG2-6	6
EG3-6	6
EG1-8	8
EG2-8	8
EG3-8	8
EG1-10	10
EG2-10	10
EG3-10	10

**Table 2:** Main characteristics of the cellular structure of EG/PU foams, density, and thermal conductivity values.

SAMPLES	Density (Kg/m^3^)	Open Cell (%)	Cell Size (µm)	NSD	AC	AR	Thermal Conductivity (mW/mK)
PU	95.9 ± 0.1	6.9 ± 0.2	467 ± 120	0.26	−0.26	1.14	48.9 ± 0.1
EG1-6	100.9 ± 1.1	9.4 ± 0.4	310 ± 105	0.34	0.61	1.26	52.7 ± 0.2
EG1-8	104.7 ± 1.7	7.8 ± 0.6	339 ± 132	0.39	0.57	1.23	53.0 ± 0.2
EG1-10	104.9 ± 0.3	9.5 ± 0.1	339 ± 108	0.32	0.56	1.25	56.0 ± 0.1
EG2-6	96.5 ± 1.7	9.4 ± 1.5	329 ± 126	0.38	0.67	1.30	50.4 ± 0.1
EG2-8	99.6 ± 0.5	9.1 ± 0.1	349 ± 121	0.35	0.85	1.04	51.6 ± 0.2
EG2-10	102.8 ± 5	9.9 ± 0.2	340 ± 104	0.31	0.83	1.14	52.8 ± 0.2
EG3-6	97.5 ± 1.5	10.8 ± 0.7	423 ± 148	0.35	0.97	1.21	51.0 ± 0.3
EG3-8	102.7 ± 0.6	9.3 ± 1.2	383 ± 181	0.47	1.12	1.12	52.0 ± 0.2
EG3-10	101.6 ± 0.9	9.5 ± 0.6	408 ± 155	0.38	1.13	1.13	52.2 ± 0.4

**Table 3:** TGA data for EG/PU samples.

SAMPLE	T_5 wt%_ (°C)	T_max_ (°C)	Residue (wt %)
PU	231	329	9.8
EG1-6	231	307	14.6
EG1-8	238	313	17.6
EG1-10	228	311	22.6
EG2-6	236	322	15.0
EG2-8	237	325	17.2
EG2-10	238	303	17.7
EG3-6	234	316	14.8
EG3-8	226	313	14.9
EG3-10	229	315	19.6

**Table 4:** Limited oxygen index (LOI) and vertical burning (UL94) tests results of EG/PU samples.

SAMPLE	EG Loading (wt %)	LOI (%)	UL94 Test	Total Burning Time (s)
PU	0	19.2	NO RATING	-
EG1-6	6	26.8	NO RATING	-
EG2-6	6	28	NO RATING	-
EG3-6	6	28.6	V-1	49.6
EG1-8	8	27.8	V-0	37.0
EG2-8	8	30	V-0	42.5
EG3-8	8	29.2	V-0	28.1
EG1-10	10	29.8	V-0	20.9
EG2-10	10	31.8	V-0	29.0
EG3-10	10	31.8	V-0	14.6

**Table 5:** Data of EG/RPUF composites at 50 kW/m^2^ from cone calorimeter test.

SAMPLE	pHRR (kW/m^2^)	THR (MJ/m^2^)	TSP (m^2^)	TSR (m^2^/m^2^)	Mass Loss (wt %)
PU	220 ± 39	68.7 ± 15.4	10.4 ± 0.4	1171 ± 42	87
EG1-6	150 ± 14	56.2 ± 4.4	7.8 ± 1.3	880 ± 149	71
EG2-6	165 ± 4	48.8 ± 0.8	4.9 ± 0.6	556 ± 76	66
EG3-6	126 ± 2	56.4 ± 3.4	3.4 ± 0.1	390 ± 5	69
EG1-8	135 ± 8	45.3 ± 1.7	3.8 ± 0.2	428 ± 26	59
EG2-8	127 ± 9	38.9 ± 3.5	2.2 ± 0.7	246 ± 81	54
EG3-8	107 ± 2	36.7 ± 19.1	1.9 ± 0.2	213 ± 17	49
EG1-10	111 ± 5	50.3 ± 7.6	2.0 ± 0.1	226 ± 1	54
EG2-10	113 ± 7	41.3 ± 0.5	2.1 ± 0.4	241 ± 43	53
EG3-10	101 ± 7	36.3 ± 0.8	1.7 ± 0.5	192 ± 54	50

**Table 6:** Mechanical data of EG/RPUF samples.

SAMPLE	Compressive Strength (MPa)	Density (Kg/m^3^)
PU	0.85 ± 0.07	95.9 ± 0.1
EG1-6	0.77 ± 0.06	100.9 ± 0.3
EG2-6	0.86 ± 0.02	97.2 ± 1.7
EG3-6	0.90 ± 0.04	97.5 ± 1.5
EG1-8	0.84 ± 0.02	104.7 ± 1.7
EG2-8	0.81 ± 0.06	101.7 ± 2.6
EG3-8	0.83 ± 0.02	102.7 ± 0.6
EG1-10	0.78 ± 0.03	104.9 ± 0.3
EG2-10	0.81 ± 0.09	102.5 ± 3.1
EG3-10	0.72 ± 0.05	101.6 ± 0.9

## Supplementary Materials

The following are available online at http://www.mdpi.com/2073-4360/11/1/168/s1.
