# Supplementary material for: Influence of the Characteristics of Expandable Graphite on the Morphology, Thermal Properties, Fire Behaviour and Compression Performance of a Rigid Polyurethane Foam
Source: Polymers (Basel). 2019 Jan 18;11(1):168. doi: 10.3390/polym11010168 (PMC6401788; doi:10.3390/polym11010168)
Supplement: Supplementary file 1 [file polymers-11-00168-s001.pdf]

Supporting information

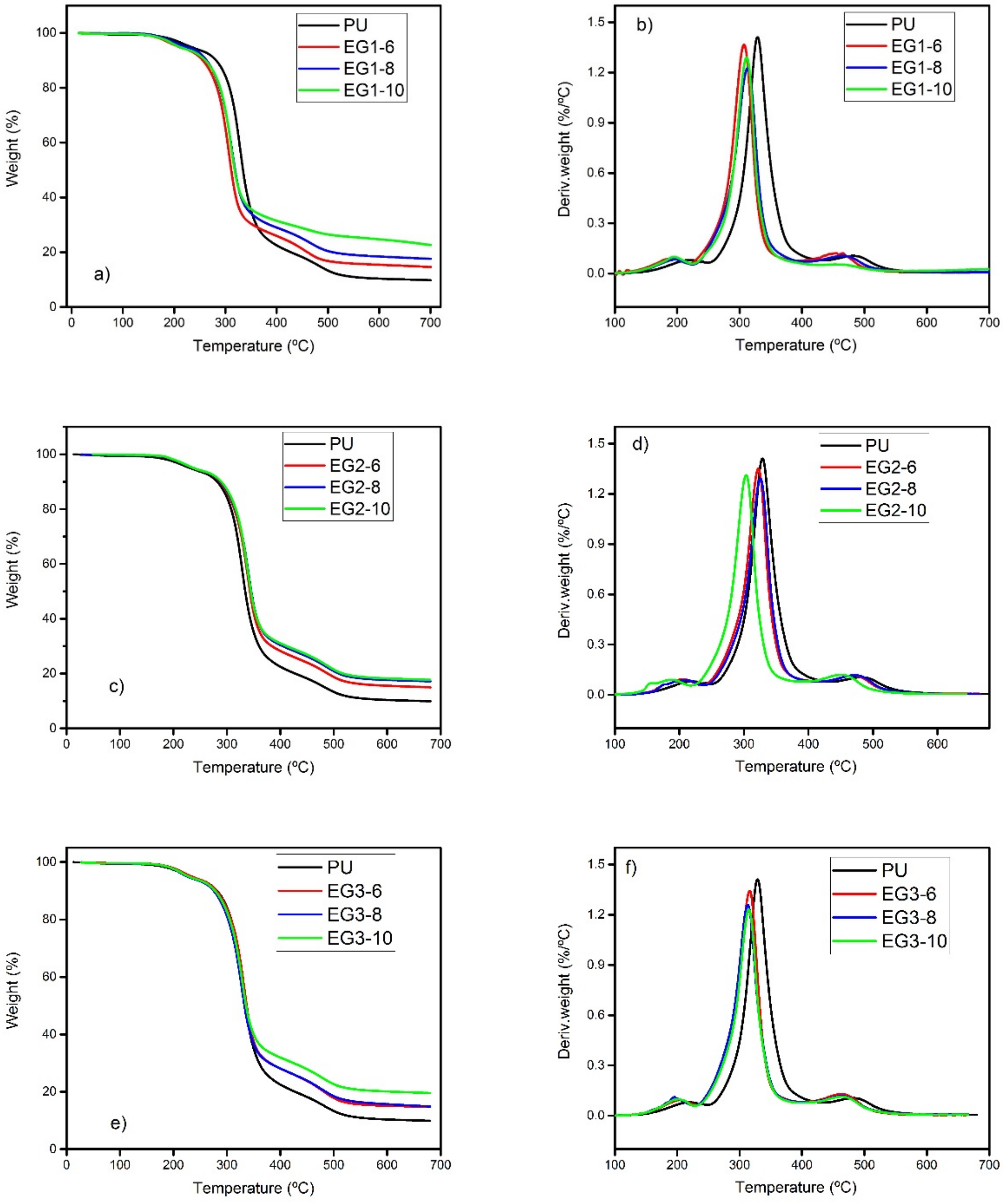

S1. TGA and DTG curves for RPUF samples containing EG1 (a) and (b), EG2 (c) and (d), and EG3 (e) and (f)

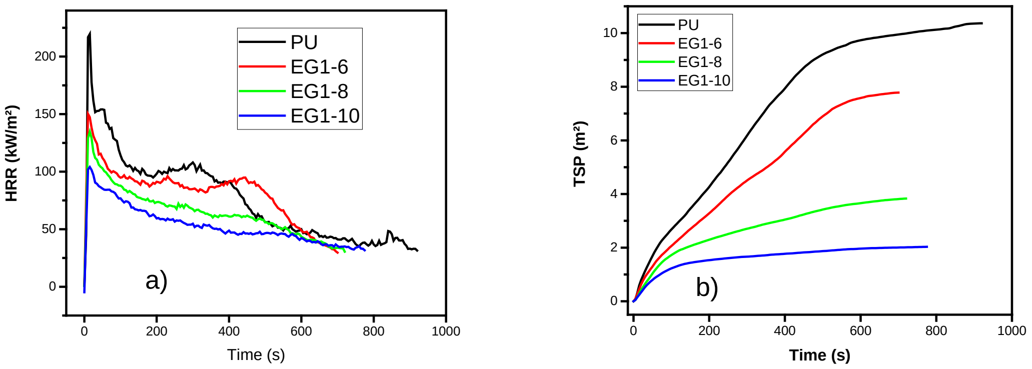

S2. a) HRR and b) TSP curves of EG1/PU samples at 50 kW/m^2

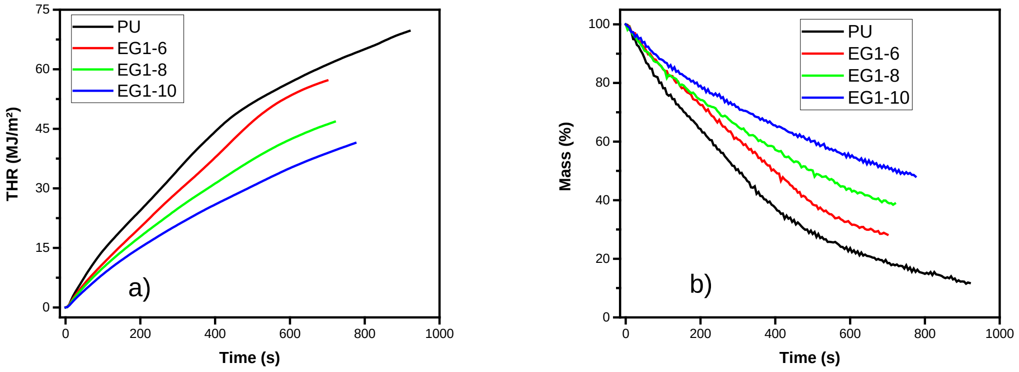

S3. a) THR and b) mass loss curves of EG1/PU samples at 50 kW/m^2

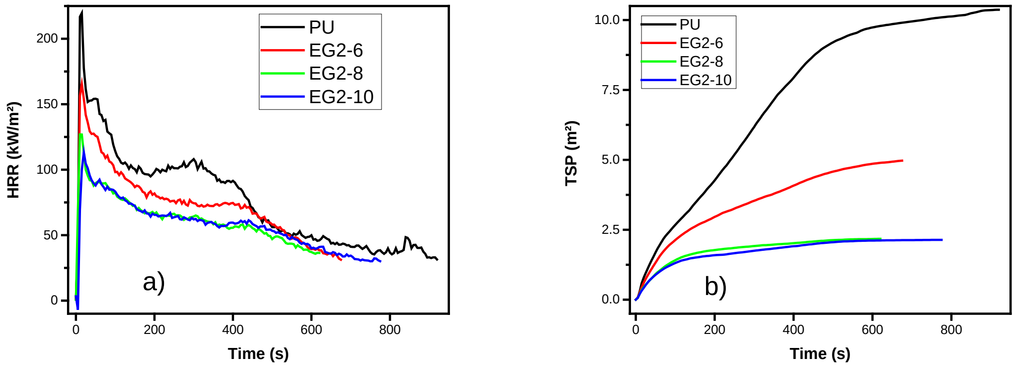

S4. a) HRR and b) TSP curves of EG2/PU samples at 50 kW/m^2

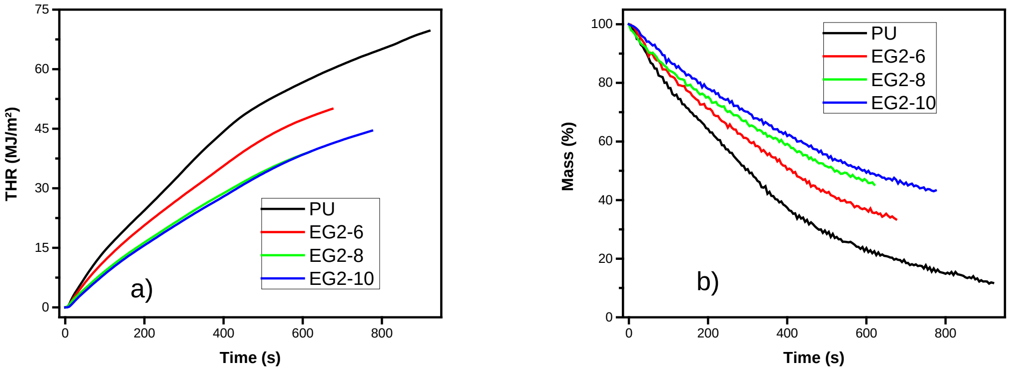

S5. a) THR and b) mass loss curves of EG2/PU samples at 50 kW/m²

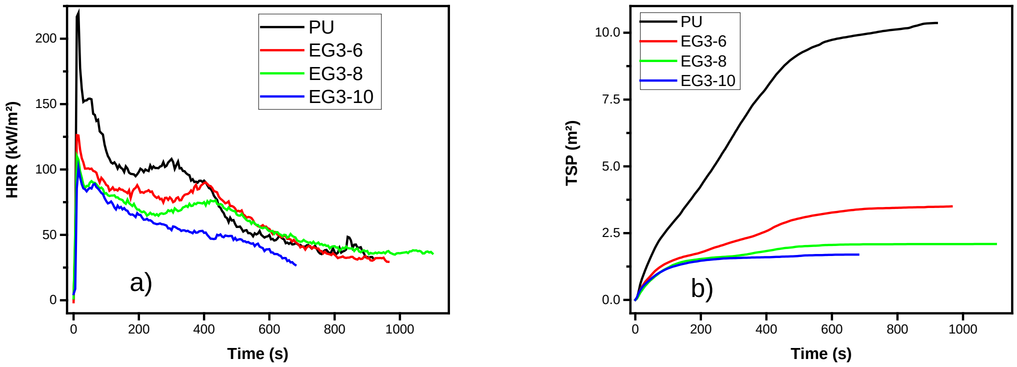

S6. a) HRR and b) TSP curves of EG3/PU samples at 50 kW/m²

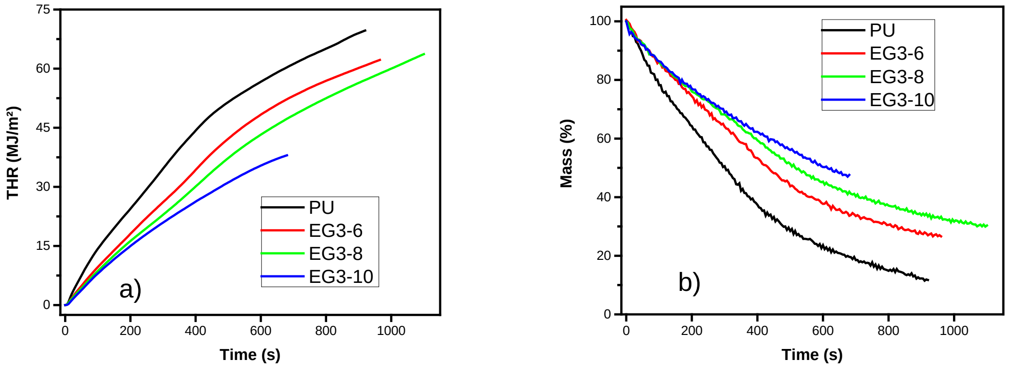

S7. a) THR and b) mass loss curves of EG3/PU at 50 kW/m²

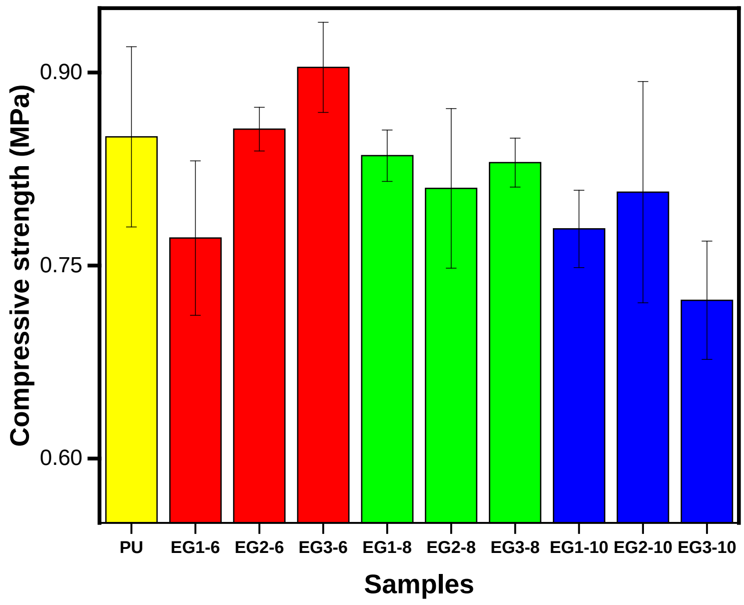

S8. Compressive strength of EG/PU samples at 3 mm/min constant rate
